# The effect of a comprehensive lifestyle intervention on cardiovascular risk factors in pharmacologically treated patients with stable cardiovascular disease compared to usual care: a randomised controlled trial

**DOI:** 10.1186/1471-2261-12-71

**Published:** 2012-09-10

**Authors:** Wilhelmina IJzelenberg, Irene M Hellemans, Maurits W van Tulder, Martijn W Heymans, Jan A Rauwerda, Albert C van Rossum, Jaap C Seidell

**Affiliations:** 1 Department of Health Sciences and the EMGO + Institute for Health and Care Research, Faculty of Earth and Life Sciences, VU University Amsterdam, De Boelelaan 1085, 1081HV, Amsterdam, The Netherlands; 2 Department of Vascular Surgery, VU University Medical Center Amsterdam, PO Box 7057, 1007MB, Amsterdam, The Netherlands; 3 Department of Cardiology, VU University Medical Center Amsterdam, PO Box 7057, 1007MB, Amsterdam, The Netherlands; 4 Department of Epidemiology and Biostatistics of the VU University Medical Center Amsterdam and the section of Methodology and Applied Biostatistics of the Faculty of Earth and Life Sciences, VU University Amsterdam, De Boelelaan 1085, 1081HV, Amsterdam, The Netherlands

**Keywords:** Cardiovascular diseases, Lifestyle intervention, Smoking, Physical activity, Diet, Health behaviour, Randomised controlled trial, Cardiology, Therapy, Cardiovascular risk management

## Abstract

**Background:**

The additional benefit of lifestyle interventions in patients receiving cardioprotective drug treatment to improve cardiovascular risk profile is not fully established.

The objective was to evaluate the effectiveness of a target-driven multidisciplinary structured lifestyle intervention programme of 6 months duration aimed at maximum reduction of cardiovascular risk factors in patients with cardiovascular disease (CVD) compared with usual care.

**Methods:**

A single centre, two arm, parallel group randomised controlled trial was performed. Patients with stable established CVD and at least one lifestyle-related risk factor were recruited from the vascular and cardiology outpatient departments of the university hospital. Blocked randomisation was used to allocate patients to the intervention (n = 71) or control group (n = 75) using an on-site computer system combined with allocations in computer-generated tables of random numbers kept in a locked computer file. The intervention group received the comprehensive lifestyle intervention offered in a specialised outpatient clinic in addition to usual care. The control group continued to receive usual care. Outcome measures were the lifestyle-related cardiovascular risk factors: smoking, physical activity, physical fitness, diet, blood pressure, plasma total/HDL/LDL cholesterol concentrations, BMI, waist circumference, and changes in medication.

**Results:**

The intervention led to increased physical activity/fitness levels and an improved cardiovascular risk factor profile (reduced BMI and waist circumference). In this setting, cardiovascular risk management for blood pressure and lipid levels by prophylactic treatment for CVD in usual care was already close to optimal as reflected in baseline levels. There was no significant improvement in any other risk factor.

**Conclusions:**

Even in CVD patients receiving good clinical care and using cardioprotective drug treatment, a comprehensive lifestyle intervention had a beneficial effect on some cardiovascular risk factors. In the present era of cardiovascular therapy and with the increasing numbers of overweight and physically inactive patients, this study confirms the importance of risk factor control through lifestyle modification as a supplement to more intensified drug treatment in patients with CVD.

**Trial registration:**

ISRCTN69776211 at http://www.controlled-trials.com

## Background

Cardiovascular disease (CVD) is a leading cause of death and loss of disability-adjusted life-years worldwide [1,2]. Because drug therapy is currently part of routine cardiovascular risk management, most patients with established CVD use cardiovascular protective medication and their cardiovascular risk profile has most likely improved.

There is strong evidence that lifestyle programmes have a beneficial effect on recurrent cardiovascular events [3-5]. Therefore, guidelines on secondary prevention and treatment for CVD emphasise the importance of lifestyle intervention [5-7]. However, the additional benefit of such interventions in a era in which most patients receive optimal treatment for hypertension, abnormal lipid profile, and disturbances in haemostatis and fibrinolysis is scarce [8,9].

The EUROASPIRE Surveys (investigating cardiovascular patients in nine countries, including the Netherlands) showed that cardiovascular disease prevention in routine clinical practice is inadequate. Lifestyle-related risk factors have deteriorated over time in these countries, and the number of overweight/obese persons with CVD is increasing [10].

Consequently there is an increasing interest in comprehensive multidisciplinary lifestyle programmes for CVD patients with multiple modifiable risk factors, who are unable to change their unhealthy lifestyle on their own [11]. However, medical staff are insufficiently trained and/or equipped to target lifestyle with such comprehensive programmes.

The EUROACTION study group showed that standards of preventive care in general hospital and general practices can be improved by a comprehensive approach that addresses all aspects of lifestyle, risk factor management, and cardioprotective drug treatments. They concluded that there was a need for local preventive cardiology programmes adapted to individual countries, which are accessible by all hospitals and general practices caring for coronary and high-risk patients [9].

Such a comprehensive multidisciplinary structured intervention aimed at lifestyle modification and maximum reduction of cardiovascular risk factors was developed in the Netherlands. This comprehensive programme (of 6 months duration) was offered in an outpatient clinic and aimed at improvement of physical activity levels, dietary habits and smoking cessation; a cardiologist adjusted cardiovascular medication after 3 months if necessary. Preliminary results from a non controlled study showed promising results [12].

To improve care in preventive cardiology, the additional benefit of risk factor control through lifestyle intervention (in addition to regular drug treatment) is important. Therefore the aim of the present study was to evaluate the effectiveness of an intensive multidisciplinary structured intervention of six months duration aimed at lifestyle modification and targeted risk factor improvement in addition to usual care in patients with CVD on cardiovascular risk factors compared to usual care alone.

## Methods

A single centre, two arm, parallel group randomised controlled trial (RCT) with a 6 month follow-up was conducted (ISRCTN 69776211, http://www.controlled-trials.com). The protocol was approved by the Medical Ethics Committee of the VU University Medical Center Amsterdam (VUmc). There were no changes to methods after trial commencement.

Patients were asked to participate in the study after receiving oral and written information, and were given time to reflect on their participation in the study before giving written informed consent. The study followed the recommendations of the Declaration of Helsinki II [13]. Data in this paper were reported in compliance with the Consolidated Standards of Reporting Trials (CONSORT) guidelines extension for parallel group randomised trials [14].

### Inclusion and exclusion criteria

Patients with stable established CVD were recruited from the vascular and cardiology outpatient departments of the VUmc. CVD was defined as coronary heart disease (CHD), angina pectoris, myocardial infarction, peripheral vascular disease, cerebrovascular accident, transient ischaemic attack, or having received surgical interventions such as a coronary artery bypass graft or percutaneous coronary intervention.

Eligible patients aged 18–75 years had to be diagnosed with CVD by a physician and had to have at least one of the following cardiovascular risk factors: hypertension (high blood pressure and/or use of antihypertensive medication), dislipidaemia, hypercholesterolaemia, or diabetes mellitus, and at least one lifestyle-related risk factor: overweight or obese indicated by a body mass index (BMI) >25, smoking, and/or physical inactivity (defined as not meeting the Dutch Physical Activity guideline) [15].

Excluded were patients with unstable CVD, a systolic blood pressure ≥ 180 mmHg and/or a diastolic blood pressure ≥ 110 mmHg, and patients with diabetes and a glycated hemoglobin (HbA_1c_) level ≥ 10.0%. Comorbidity had to be stable and be judged by the referring physician as no contraindication to engage in physical exercise. Finally, patients should be able to climb stairs and adequately communicate in the Dutch language.

Eligible patients were informed by their medical specialist and invited to participate. They received an information package containing a letter of invitation, a study leaflet, a questionnaire, and a return envelope. Additional potentially eligible patients were identified from the database of the departments of cardiology and vascular surgery. These patients were contacted by telephone to verify their eligibility shortly after they received the information package by mail. If the patient was willing to participate their specialist and general practitioner (GP) were informed. Written informed consent was obtained from all participants at the time of enrolment.

### Randomisation and blinding

An independent researcher (not involved in the selection/treatment of patients) prepared the randomisation with a computer-generated digital table of random numbers in blocks of 8. To ensure blinding of treatment allocation, a research nurse randomly assigned patients to either the intervention or control group using an on-site computer system combined with allocations kept in a locked computer file.

Caregivers could not be blinded for treatment allocation; however, apart from evaluation of physical capacity (using strict objective criteria and standard protocols) they were not involved in the assessment of outcome measurements. Also, the research nurse performing the physical measurements could not be blinded for treatment allocation; however, she was not involved in the intervention and this could not have affected any of the more objective outcome measurements e.g. laboratory results. The principal investigator was blinded for the allocation of the intervention when performing the data analysis. For obvious reasons, patients could not be blinded for treatment allocation. Patients allocated to the control group were informed that they could participate in the intervention after the study had finished.

### Interventions

The intervention group received the comprehensive multidisciplinary structured lifestyle intervention, in addition to usual care.

#### Intervention programme

The lifestyle programme was developed by a team consisting of a physiotherapist, sport physician, cardiologist, psychologist, nutritionist and internist [12]. The programme was offered in a specialised outpatient clinic and was targeted at optimal cardiovascular risk factor reduction by dietary modification, physical exercise and smoking cessation. If necessary, additional medical therapy could be provided after the first 3 months, targeted at optimal risk factor reduction.

Prior to start of the lifestyle programme, a diagnostic programme took place. A standardised computer questionnaire on medical history, symptoms, lifestyle and medication formed the basis of a detailed medical history. Standard measurements of body height/weight and blood pressure were made. Blood samples were taken for determination of total/HDL/LDL cholesterol levels. Finally, a physician performed a physical examination and maximal work rate was measured on a bicycle ergometer. Patients were given the results by the physician, and a written medical report focusing on risk factors and risk behaviour was handed to the patient and mailed to the referring physician. The same diagnostic programme was repeated 6 months later.

The lifestyle programme lasted 6 months. In short, the first 3 months focused on intensive group-based (maximum 12 persons) physical training, education and counselling. The exercise programme was offered twice weekly, consisting of an individualised exercise session of 1 h (based on aerobic threshold and maximal heart rate) followed by a relaxation session of 30 min, giving a total of 22 sessions in 3 months. During this period a group counselling programme consisting of 7 sessions focusing on risk factors, physical activity, diet, motivation, stress management and training modalities was supervised by one of the specialised team members. During the last session, patients were instructed to design a personal training programme to incorporate a long-term maintenance regimen in daily life using support systems such as a gymnasium or other facilities of their choice.

In the second 3 months, the aim was to implement lifestyle changes in everyday life, supported by a monthly exercise session led by the physiotherapist. All patients had to be referred by a physician to be eligible for compensation from their health insurance company, leaving a relatively small financial contribution to be made by patients allocated to the lifestyle programme.

Smokers were encouraged to quit and offered assistance from a psychologist who conducted an individually-tailored psychosocial intervention. Patients had to set a target date to stop smoking, and follow-up visits were scheduled to monitor progress. A nutritionist informed patients about a low-fat diet. The dietary recommendations consisted of a mixed low-fat and Mediterranean regimen [16,17].

#### Control group

Usual care or conventional risk-factor treatment was provided by the GP or medical specialist according to the Dutch guidelines for the care of patients with CVD [7,18]. In the Netherlands, patients with CVD are treated with blood pressure-lowering medication, lipid-lowering medication (e.g. statins), anticoagulants, ACE inhibitors, beta-blockers, antiplatelet therapy, and antithrombotics. In addition, patients with diabetes receive oral glucose-lowering medication and/or insulin. Usual healthy lifestyle advice is given.

### Compliance and co-interventions

The number of sessions attended was registered, and compliance was considered adequate if at least 15 of the 22 exercise sessions (70%) were attended. No patients were restricted in their options to obtain additional health care. During the intervention period and follow-up, co-interventions were registered and evaluated in both groups.

### Outcome assessment

The primary outcome measures were the lifestyle-related cardiovascular risk factors (smoking status, exercise and eating habits), and other cardiovascular risk factors including weight, BMI, waist circumference, diastolic/systolic blood pressure, total/HDL/LDL cholesterol, physical fitness and changes in medication. Secondary outcome measures were estimated risk of cardiovascular morbidity, total mortality, and quality of life.

### Data collection

Data on physical measurements and self-administered questionnaires were collected at baseline, and at 3 and 6-months follow-up. A research nurse performed the physical measurements and collected the questionnaires.

#### Anthropometric measurements and laboratory tests

Physical measurements were made according to a standardised protocol. Individuals who did not attend the 3-month follow-up were contacted twice (by mail and by telephone). Those who did not withdraw from the study were considered ‘not available’ for that follow-up and were contacted for the next follow-up.

Body weight/height were measured to calculate BMI. Height was measured to the nearest 0.1 cm without shoes, and weight to the nearest 0.5 kg wearing underwear and no shoes. Persons with a BMI ≥ 25 and < 30 were considered overweight, those with a BMI of ≥ 30 were considered obese. Waist circumference was measured following the WHO criteria [19]. Systolic/diastolic blood pressure was measured in a standard way (Omron 705IT): the first measurement was made after 5 min rest in sitting position and the mean of the three measurements per visit was used.

Blood and urine samples for determination of total/HDL/LDL cholesterol, glucose, and Hb1Ac were collected after overnight fast.

Maximal work rate was measured on a bicycle ergometer and the corresponding metabolic equivalent of task (MET) scores were calculated [20].

#### Questionnaires

On three occasions (with a 3-month interval in between) patients completed a self-administered questionnaire on (family) medical history, lifestyle, depression, general health status and health-related quality of life (HRQL). Questionnaires were either given at the recruitment visit, or sent home with the request to bring the completed questionnaire to the next visit. Additional information on medical history was retrieved from the patient’s medical file.

At baseline, various prognostic measures were collected to evaluate whether randomisation successfully resulted in two groups with comparable prognosis, and (if necessary) to adjust for baseline differences in the analyses. Sociodemographic information included age, sex, level of education, ethnicity and working status.

The validated Short Questionnaire to Assess Health-enhancing physical activity (SQUASH) questionnaire was used to assess self-reported levels of physical activity [21]. Patients were asked to refer to an average week in the past few months. Using the Ainsworth compendium of physical activities [22], activities were assigned a MET value. One MET is defined as the energy expenditure for sitting quietly. Based on the Dutch physical activity guideline [15], activities were subdivided for adults and older adults (up to age 55 and older) respectively into three intensity categories. The total amount of minutes per day that a patient was performing light (2–4MET), moderate (age > 18–55 years: 4–6.5 MET; age >55 years: 4–5 MET), or heavy physical activity (age 18–55 years ≥ 6.5 MET; age >55 years: ≥ 5 MET) was calculated (MET = unit of metabolic equivalent, which is the ratio of the energy cost of a given activity to resting metabolic rate and was derived from published tables [22].

Eating behaviour was assessed by a Dutch Food Frequency Questionnaire (DFFQ) [23]. A smoker was defined as someone who reported to smoke at least one cigarette, cigar or pipe in the past week. Abstinence was assessed by self-report; the outcome is dichotomous (abstinent versus smoking).

General health was measured with the SF-36, which has a good reliability and validity [24]. It yields a physical component and mental component; both scores range from 0 to 100, with higher scores representing better health. Level of depressive symptoms was measured using the 20-item Centre for Epidemiologic Studies Depression Scale (CES-D) [25]. Total scores range from 0 to 60. A cut-off score of ≥ 16 was used to identify respondents with a clinically significant level of depression [26].

HRQL is considered to be an integral part of outcome measures in preventive cardiology [27]. The MacNew is a disease-specific instrument for measuring HRQL in cardiac patients; it is valid and reliable to measure HRQL of patients with CHD [28-30]. Scores range from 1–7, with higher scores indicating a better HRQL [28]. The VascuQol is a disease-specific HRQL instrument for peripheral arterial disease. Patients’ responses are converted to a scale ranging from 1 (worst possible score) to 7 (best possible score) [31]. A total score is calculated, with a higher score indicating a better HRQL. The 10-year risk of developing cardiovascular events and mortality was estimated using the Copenhagen Risk Score (PRECARD risk profile) [32].

### Statistical analysis

In this study we aimed to study the cost-effectiveness. We had based our sample size calculation on finding a difference in total costs between the two groups of 15%. However, we had to stop recruitment prematurely due to a lack of financial resources. During the recruitment period there was a change in insurance policy. The costs of the intervention were no longer reimbursed for patients. In the end we had 71 patients in the intervention group and 75 in the control group. The primary outcome measure used in the power calculation of this study was the Copenhagen Risk Score (CRS). We used a European risk calculation programme named PRECARD to calculate the absolute risk of CVD. The CRS is based on age, gender, BMI, history of CVD, Diabetes Mellitus, smoking status, systolic blood pressure, HDL and total cholesterol levels. A post-hoc power calculation showed that in order to detect a difference in change of 5% in absolute estimated CVD risk calculated by the CRS with a standard deviation of 10%, with statistical significance (alpha = 0.05) and sufficient power (1-beta = 0.80) we needed 62 patients in each group. With a possible drop-out rate of 15% the number of participants to be randomised was 72 per group.

The effects of the intervention on outcome measures at 3 and 6 months follow-up were analysed according to the intention-to-treat principle [33], including all subjects regardless of whether or not they actually received the complete intervention. The analysis was conducted with all available respondents at the time of follow-up; non-response analyses were conducted to evaluate whether drop-out during the first or second follow-up period was associated with health status or intervention status. The effects of the intervention on the continuous health measures were evaluated with linear regression models. All regression models were adjusted for sex and age, and included the baseline value of the health measure of interest. Resulting regression coefficients can be interpreted as the difference in patient outcomes between both groups at a certain follow-up period corrected for the difference at baseline. The effects of the intervention on the dichotomous health measures were analysed by logistic regression. In a sensitivity analysis, the multiple imputations technique was used for missing data.

Analyses were performed with the statistical package SPSS.

## Results

### Participants

**Figure 1:**
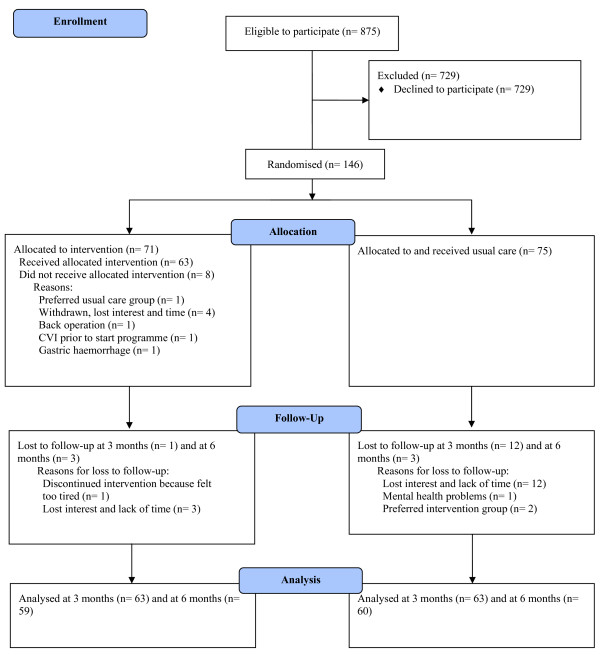
Flow diagram of the progress of participants through the trial.

Between September 2005 and February 2007, 146 participants were enrolled in the study. Figure 1 presents the CONSORT diagram [34] of the flow of participants through the phases of the trial. At baseline, 875 patients were invited to participate of which 146 (17%) gave written consent and completed baseline measurements. Patients with a low level of education and retired patients were less likely to participate. Motives and barriers underlying the decision to participate or not were explored and reported elsewhere [35].

A total of 71 patients were assigned to the intervention programme and 75 to the usual care group; 82% of the patients remained in the study during the 6-month follow-up. Analyses showed no selective loss to follow-up. Table 1 presents the baseline characteristics of the study population. Baseline characteristics of the outcome measures are presented in Tables 2, 3, 4 and 5. The randomisation was successful in creating study groups with largely similar baseline values, including current and past health conditions. Therefore, adjustment was made only for the baseline values of the outcome measures sex and age, and not for the other prognostic variables. Smoking status showed a difference at baseline and was corrected for in the analyses when necessary.

**Table 1:** Baseline characteristics of the study population (n = 146)

	**Interventionn = 71**	**Controln = 75**
**Sociodemographic characteristics**		
Mean age*, years*	60.4 (13.1)	59.6 (8.4)
Gender male, *n* (%)	60 (84.5)	53 (70.7)
Education Lower education, *n* (%)	20 (28.6)	27 (37.0)
Intermediate education, *n* (%)	32 (45.7)	27 (37.0)
Higher education, *n* (%)	18 (25.7)	19 (26.0)
Marital or cohabitation status, *n* (%)	49 (69.0)	54 (72.0)
Working status: Paid job, *n* (%)	23 (32.4)	28 (37.3)
Ethnic minority, *n* (%)	8 (11.3)	12 (16.0)
**Prognostic factors**		
**Clinical characteristics of CVD**		
Cardiology patients, *n* (%)	60 (84.5)	63 (84.0)
NYHA I ^a^	34 (47.9)	30 (40.0)
NYHA II ^a^	24 (33.8)	30 (40.0)
NYHA III ^a^	2 (2.8)	3 (4.0)
Vascular patients, *n* (%)	11 (15.5)	12 (16.0)
PAV I ^b^	4 (5.6)	5 (6.7)
PAV II ^b^	6 (8.5)	6 (8.0)
PAV III ^b^	1 (1.4)	0 (0.0)
Angina Pectoris, *n* (%)	6 (8.5%)	5 (6.7)
Myocardial Infarction, *n* (%)	58 (81.7)	62 (82.7)
Coronary Artery Bypass Graft, *n* (%)	26 (36.6)	23 (30.7)
Percutaneous Coronary Intervention, *n* (%)	31 (43.7)	38 (50.7)
Cerebrovascular Accident, *n* (%)	9 (12.7)	4 (5.3)
Comorbidity Diabetes Mellitus, *n* (%)	18 (25.4)	14 (18.7)
At risk for depression (CES-D score ≥ 16), *n* (%)	19 (26.8)	20 (26.7)
Family history of CVD	31 (43.7)	34 (45.3)

Numbers are mean (SD) unless stated otherwise.

CVD = cardiovascular disease; ^a^NYHA category among cardiology patients; ^b^PAV category among vascular patients.

**Table 2:** Prescribed medication at baseline and at 6-months follow-up for those who remained in the trial (n = 146)

**Drug class**	**Baseline data for those who remained in the trial**		**Data at 6-months follow-up**	
	**Intervention n = 59**	**Control n = 60**	**Intervention n = 59**	**Control n = 60**
**Antithrombotica**				
Acetylsalicylic acid (aspirin)	47 (79.7%)	54 (90.0%)	49 (83.1%)	52 (86.7%)
**Blood pressure-lowering medication**				
Angiotensin-converting enzyme (ACE inhibitors)	24 (40.7%)	21 (35.0%)	27 (45.8%)	20 (33.3%)
β-blocker	34 (57.6%)	38 (63.3%)	33 (55.9%)	40 (66.7%)
Calcium-channel blocker	12 (20.3%)	19 (31.7%)	16 (27.1%)	18 (30.0%)
Diuretica	12 (20.3%)	12 (20.0%)	12 (20.3%)	10 (16.7%)
**Lipid-lowering medication**				
Statins	51 (86.4%)	49 (81.7%)	53 (89.8%)	54 (90.0%)
**Blood glucose-lowering medication**^**a**^				
Biguanides	9 (15.3%)	7 (11.7%)	10 (16.9%)	7 (11.7%)

n=number of patients; %, percentage of patients who remained in the study; ^a^data based on diabetic patients at baseline and at 6-months follow-up.

**Table 3:** Differences in minutes per day spent on different categories of physical activity (mean ± SD), eating and smoking behaviour between patients in the intervention (n = 71) and control group (n = 75) at baseline, and at 3 and 6 months follow-up

**Primary outcome measure**	**Baseline**		**3-months follow-up**		**6-months follow-up**	
	**Intervention n = 71**	**Control n = 75**	**Intergroup differences**^**a**^**B(95% CI)**	***p***	I**ntergroup differences**^**a**^**B(95% CI)**	***p***
**Physical activity** SQUASH, *min/week*						
Moderate intensity	43.9 (75.3)	33.9 (62.9)	8.7 (−128.5/145.8)	0.90	187.2 (23.4/351.0)**	0.03
Heavy intensity	31.0 (60.2)	30.1 (57.7)	121.8 (−12.1/255.6)*	0.07	−4.2 (−123.0/114.6)	0.95
At least moderate (moderate + heavy)	74.9 (100.3)	64.1 (85.1)	112.4 (−92.8/317.6)	0.3	189.6 (−1.0/380.2)**	0.05
**Eating behaviour** DFFQ						
Average vegetable intake/day, *grams*	172.4 (91.9)	181.9 (90.1)	−2.0 (−27.3/23.2)	0.98	−0.5 (−25.7/24.7)	0.97
Number of fruit intake/day	1.5 (1.3)	1.7 (1.2)	0.03 (−0.3/0.3)	0.86	−0.05 (−0.4/0.3)	0.8
Number of days breakfast	6.3 (1.8)	5.5 (2.6)	0.07 (−0.4/0.6)	0.80	0.5 (−0.1/1.1)*	0.09

* p < 0.1; **p < 0.05.

**Table 4:** Data on mean (SD) primary outcome measures at 3 and 6-months follow-up in the intervention group (n = 71) and control group (n = 75) and the estimated effect of the multidisciplinary lifestyle intervention on cardiovascular risk factors compared with the control group

**Primary outcome measure**	**Baseline**		**Baseline to 3 months**		**6-months**	
	**Intervention, n = 71 mean (SD)**	**Control, n = 75 mean (SD)**	**Intergroup differences**^**a**^**B(95% CI)**	***p***	**Intergroup differences**^**a**^**B(95% CI)**	***p***
Weight, *kg*	86.0 (15.0)	88.0 (20.6)	−1.3 (−2.2/-0.4)** ^b^	0.004	−1.3 (−2.5/-0.1)**^b^	0.03
Body mass index, *kg/m*^*2*^	28.7 (4.4)	29.8 (5.4)	−0.5 (−0.8/-0.2)** ^b^	0.002	−0.5 (−0.9/-0.1)**^b^	0.02
Waist circumference, *cm*	101.2 (13.4)	101.0 (14.7)	−2.5 (−4.0/-1.0)**	0.001	−0.2 (−2.0/1.6)**	0.82
Systolic BP, *mmHg*	137.8 (20.5)	140.0 (18.2)	−3.6 (−7.9/0.7)	0.10	−2.7 (−8.0/2.5)	0.30
Diastolic BP, *mmHg*	80.2 (10.1)	79.3 (10.5)	−1.7 (−4.0/0.6)	0.15	−1.0 (−3.9/1.8)	0.47
Total cholesterol, *mmol/l*	4.2 (0.8)	4.6 (1.1)	−0.1 (−0.3/0.2)	0.55	−0.2 (−0.5/0.1)	0.25
HDL cholesterol, *mmol/l*	1.3 (0.3)	1.4 (0.4)	0.02 (−0.1/0.1)	0.68	−0.0001 (−0.1/0.1)	0.99
Cholesterol/HDL ratio	3.3 (0.9)	3.4 (1.1)	0.06 (−0.2/0.3)	0.61	−0.1 (−0.4/0.2)	0.58
LDL cholesterol, *mmol/*l	2.2 (0.8)	2.4 (1.0)	0.001 (−0.2/0.2)	0.99	−0.1 (−0.3/0.2)	0.61
HbA_1c_,*%*^1^	7.1 (1.8)	7.1 (1.3)	−0.3 (−0.9/0.4)	0.38	−0.5 (−1.2/0.2)	0.14
Maximal work rate,*Watt*	146.1 (43.5)	156.4 (49.4)	6.9 (0.9/13.0)**	0.03	n.a.	
Maximal ergometry, *METS*	6.4 (1.6)	6.7 (1.8)	0.3 (0.1/0.5)**	0.01	n.a.	

^a^Adjusted for age, sex, baseline value of outcome measure and ^b^if appropriate also adjusted for smoking; B-values reflect differences between groups over time from baseline. CI = confidence interval; BP = blood pressure; HDL = ‘high density lipoprotein’; LDL = ‘low density lipoprotein’; HbA_1c_ = glycolysed haemoglobin; n.a. = not applicable.

^1^only among patients with diabetes mellitus type 2; **p < 0.05.

**Table 5:** Effect of the multidisciplinary structured lifestyle intervention on estimated 10-year risk of cardiovascular events, general health and quality of life among patients with stable cardiovascular disease compared with usual care at baseline, and at 3 and 6 months

**Secondary outcome measures**	**Baseline**		**3 months**		**6-months**	
	**Intervention n = 71**	**Control n = 75**	**Intergroup differences**^**a**^**B(95% CI)**	***p***	**Intergroup differences**^**a**^**B(95% CI)**	***p***
**10-year risk cardiovascular event**^**a**^						
Coronary heart disease	37.3 (20.3)	33.8 (18.8)	−0.6 (−3.4/2.2)	0.67	−0.6 (−4.3/3.1)	0.75
Myocardial infarction	18.9 (13.9)	17.1 (11.6)	−1.1 (−3.0/0.9)	0.29	−0.8 (−3.5/1.8)	0.53
Cerebrovascular accident	8.2 (8.0)	7.3 (6.1)	−0.5 (−1.5/0.4)	0.26	−0.3 (−1.4/0.9)	0.66
Total mortality	35.0 (18.1)	34.1 (17.1)	0.2 (−2.6/3.0)	0.89	1.1 (−1.6/3.8)	0.43
**General health and quality of life**						
SF-36 MCS	52.9 (9.8)	52.2 (11.0)	0.6 (−1.6/2.9)	0.58	−0.8 (−3.1/1.5)	0.50
PCS	39.6 (10.6)	42.2 (9.5)	1.6 (−1.0/4.1)	0.23	1.0 (−1.7/3.7)	0.47
MacNew ^b^	5.3 (0.8)	5.2 (0.9)	0.06 (−0.1/0.3)	0.56	0.04 (−0.2/0.2)	0.69
Vascuqol ^c^	4.8 (1.4)	5.6 (0.9)	−0.09 (−0.9/0.7)	0.81	−0.2 (−1.7/1.3)	0.78

Numbers are mean (SD) unless stated otherwise; 95% CI= 95% confidence interval; MCS=^,^ norm-based Mental Composite Score; PCS=norm-based Physical Composite Score; ^a^Using the PRECARD®-riskscore^24^; ^b^total score only among cardiology patients; ^c^total score only among vascular patients.

### Compliance

Compliance with the programme was good; on average 18 (range 2–22) of the 22 (80%) physical exercise sessions were attended. One person stopped after two sessions having suffered a myocardial infarction on vacation.

### Effect on prescribed medication

At baseline all patients in both groups received medication for CVD. Table 2 shows that the prescribed medication was almost identical in the two groups during follow-up.

Most patients received aspirin, beta-blockers and statins. Overall, during the 6-month follow-up, changes in prescribed medication were made in 35.6% (n = 21) of the intervention group and in 30.0% (n = 18, including additional gastro-medication in 1 patient) of the control group. There were no intergroup differences for the use of any specific drug.

### Effect of the intervention on primary outcome measures

#### Lifestyle-related cardiovascular risk factors

Table 3 shows the effect of the intervention on lifestyle measures. The intervention group showed a significant increase in physical activity level per day compared with the control group. There were no important differences in eating behaviour. After 6 months the intervention group was found to consume 0.5 more servings of breakfast per week than participants receiving usual care (p < 0.1). Favourable fat use was similar in both groups.

Smokers in the intervention and control group had smoked for (mean) 29.5 (SD 18.3) years and 33.0 (16.3) years, respectively. The intervention was not successful in changing smoking behaviour. In the intervention group 2 of the 10 smokers for whom there was a follow-up available had quit smoking, while one non-smoker started smoking again. In the control group none of the smokers quit smoking.

#### Other modifiable cardiovascular risk factors

Table 4 presents the effects on other primary outcome measures. Weight, BMI and waist circumference showed a significant decrease in the intervention group compared with the control group. There were no significant differences at 3 and 6 months in mean blood pressure and total/HDL/LDL levels. There was a significant increase in maximal work rate in the intervention group compared with the control group (6.9 Watt [95% CI: 0.9/13.0]; 0.3 METS [95% CI: 0.1/0.5]). These results remained largely the same after additional adjustment for smoking status.

### Effect of the intervention on secondary outcome measures

Table 5 presents the effects of the programme on secondary outcome measures. There was no significant difference in estimated 10-year risk for cardiovascular morbidity and mortality, general health, and HRQL between the two groups.

### Sensitivity analysis

In the sensitivity analyses no effect of the intervention was found for specific subgroups of patients who reported depression. Multiple imputations for missing data showed similar effects of the intervention on all outcome measures.

## Discussion

This study shows that even in intensely pharmacologically-treated CVD patients who received cardioprotective drug treatment, a comprehensive lifestyle intervention had a beneficial effect on some cardiovascular risk factors. The intervention decreased BMI and waist circumference, and increased exercise capacity and levels of moderate/heavy physical activity. There was no added significant effect on any other outcome measure.

The results achieved with this multidisciplinary structured lifestyle intervention programme of improvement in physical activity, BMI and exercise capacity are in line with other intensive lifestyle programmes for patients with a history of CVD [36-38]. For instance the EUROACTION preventive cardiology programme, that was quite similar to the intervention in this study, also led to some weight loss and for high risk patients in a reduction of central obesity. No significant effects were found on lipid levels. The programme also improved blood pressure control without the use of additional antihypertensive drugs [9]. However in our study setting, cardiovascular risk management for blood pressure and lipid levels by prescription of medical prophylactic treatment for CVD in usual care was close to optimal (as reflected in baseline levels); therefore, the additional benefit of the lifestyle intervention over usual care for blood pressure and lipid levels was small and not significant. Even though the smoking cessation strategy used in the present study was evidence based, few patients ceased smoking; this might be due to the small numbers involved. In a Cochrane review the overall effect of psychosocial smoking cessation interventions in CHD patients was expressed by a number needed to treat of 9.7 [39]. This means that about 10 patients had to be treated for one person to have abstained from smoking after 1 year. Another explanation could be the relatively high percentage of heavy smokers in our study population. Similarly, other studies found a higher prevalence of heavy smokers in those who continue to smoke or who relapse after a cardiac event [40-42]. It remains unclear which method is most effective to help these patients stop smoking [40,42].

There was no effect on estimated 10-year risk of cardiovascular morbidity and total mortality. One reason could be the latency of effects, i.e. benefits might not be detected in the early stages but may emerge over time. The present study may have been too short to show an impact on morbidity and mortality. Another reason could be that the individual risk assessment was performed using the Copenhagen Risk Score [32]; although this score is suitable for many of the parameters in the present study, it does not allow input of exercise capacity as an independent risk factor. The positive effect of exercise on cardiovascular morbidity and mortality has been well documented [43]. Myers et al. reported that exercise capacity was a stronger predictor of mortality than other established risk factors in men with a history of CVD [44]. Manson et al. [45] reported that both walking and vigorous exercise are associated with substantial reductions in the incidence of cardiovascular events irrespective of age and body mass index. Thus, in the present study, the calculated risk score probably underestimated the protective effect of the lifestyle intervention.

### Methodological limitations

The strength of the study is its randomised design which reduces the chance of confounding.

A limitation is that all patients were receiving cardiovascular medication at baseline, and medication use for patients that successively altered their lifestyle was not lowered. Therefore, we could not prove the additional benefit of the intervention over usual care for blood pressure and lipid levels. However, nowadays it is not feasible to recruit patients with established CVD that are not on cardiovascular medication and (of course) it is unethical to stop providing such medication to patients randomised to the intervention group.

A potential limitation of the external validity of this study is the non-response of patients eligible to participate. Participants were higher educated, slightly younger, and more often still at work compared with non-responders. Also, patients participating in a trial may be more amenable to behavioural change than those declining participation. Therefore, our conclusions may not apply to patients with a lower education level or those less motivated to change their lifestyle.

In this study we had problems with the recruitment of patients. The main reason was that eligible patients refused to participate. Low participation rates for secondary prevention programmes have been reported repeatedly [46]. Another reason was the lack of financial resources. During the recruitment period of the study there was a change in policy of the health insurance companies. Consequently, the intervention was no longer reimbursed for patients and we had to stop recruitment. A post-hoc power calculation showed that we were still able to assess the effectiveness of the programme on clinical outcomes.

The present findings may have implications for policy on preventive cardiology for patients with established CVD and for future research.

First, the results show that the benefits of lifestyle intervention are sustained in the present era of widespread cardiovascular therapy. Moreover, there is a growing population of overweight, obese and physically inactive CVD patients [10]. These trends cannot be effectively targeted with medication alone and this trial confirms the benefits of lifestyle modification on these risk factors. Our results are an encouraging sign that preventive cardiology can be further improved. An economic evaluation is needed to determine the cost-effectiveness of the intervention.

Current evidence shows the benefits of a wide variety of secondary prevention programmes including less intensive and shorter versions [4,47]. In the Netherlands distances to clinics are relatively short. For many countries and rural areas less intensive interventions may be a good alternative, because they are easily accessible and less costly. However, this may not extend to a clinically more complicated older, fragile population that tends to be more complex in terms of co-morbidities and treatment regimen. They may need close supervision [3]. Future research should investigate the effectiveness and cost-effectiveness of less intensive interventions compared with more intensive interventions.

Second, we recommend that future strategies for cardiovascular risk reduction should begin with lifestyle modification and introduce or lower prophylactic cardiovascular medication later on if required. Lifestyle modifications avoid the adverse effects associated with medication and are less costly than long-term medication.

Third, a large trial is needed to compare usual care with an intervention aimed at lifestyle modifications together with the active lowering of medication.

## Conclusions

Even in CVD patients receiving good clinical care and using cardioprotective drug treatment, a comprehensive lifestyle intervention had a beneficial effect on some cardiovascular risk factors. In the present era of cardiovascular therapy and with the increasing numbers of overweight and physically inactive patients, this study confirms the importance of risk factor control through lifestyle modification as a supplement to more intensified drug treatment in patients with CVD.

## Abbreviations

CVD: Cardiovascular disease; RCT: Randomised controlled trial; VUmc: VU University Medical Center; CHD: Coronary heart disease; BMI: Body mass index; HbA_1c_: Glycated hemoglobin; GP: General practitioner; MET: Metabolic Equivalent of Task; HRQL: Health-related quality of life; SQUASH: Short Questionnaire to Assess Health-enhancing physical activity questionnaire; DFFQ: Dutch Food Frequency Questionnaire; CES-D: Centre for Epidemiologic Studies Depression Scale; PRECARD risk profile: CRS, Copenhagen Risk Score.

## Competing interests

The authors declare that they have no competing interests with submission of this manuscript.

## Authors‘ contribution

JS obtained funding, conceived and designed the study, and critically revised the manuscript. WIJ made the study operational and supervised data collection, analysed and interpreted the data, and drafted the manuscript. IH supervised the medical study and safety of patients, and critically revised the manuscript. MH provided statistical assistance. JR and BR, MT cooperated in the recruitment of study patients, and critically revised the manuscript. All authors read, edited and approved the final manuscript.

## Pre-publication history

The pre-publication history for this paper can be accessed here:

http://www.biomedcentral.com/1471-2261/12/71/prepub
